# Conclusions of the SIGNAL study in Huntington and implications for treatment of other slowly progressive neurodegenerative diseases

**DOI:** 10.1002/ctm2.1169

**Published:** 2023-01-29

**Authors:** Maurice Zauderer, Elizabeth Erin Evans

**Affiliations:** ^1^ Vaccinex, Inc. Rochester New York USA

**Keywords:** neurodegeneration, pepinemab, semaphorin 4D, SEMA4D

Astrogliosis is associated with slowly progressive neurodegenerative/neuroinflammatory diseases, including Huntington's (HD) and Alzheimer's (AD). In their normal homeostatic state, astrocytes extend numerous specialised cytoplasmic projections that fully cover brain capillaries and facilitate glucose uptake while also extending projections to synapses that recycle 80% of released excitatory transmitters.^1^ However, under conditions of disease‐related stress, they undergo a dramatic transformation in which their actin cytoskeleton collapses, they retract cytoplasmic projections, build up secretory machinery, and switch to synthesis and secretion of soluble biological effectors some of which have neurotoxic activity and others recruit and activate other inflammatory cells.^2^

In two recent publications,^3^ ^.^ ^4^ we investigated the signals that trigger the striking morphological transformation of astrocytes. Based on the previous work of our own and others, we were aware of the important role of the semaphorin 4D:plexin‐B1/B2 pathway in triggering collapse of the actin cytoskeleton. Binding of Semaphorin 4D (SEMA4D) ligand to the extracellular domain of plexin‐B receptors triggers activation of their cytoplasmic Rho‐GTPase activating domain. This leads to the inactivation of Rho kinase which is normally required to initiate a chain of events that prevents cofilin‐driven depolymerisation of actin filaments.^5^ We, therefore, investigated expression of SEMA4D and its receptors under disease conditions in neurons and astrocytes.^4^ We made the following observations:

1. SEMA4D staining of post‐mortem sections from normal human control and from people with HD and AD demonstrated significantly increased SEMA4D expression in neurons during disease progression in brain regions of three different patients at each of HD pathological stages 0, 1, and 2 as well as in advanced AD.
2. plexin‐B1 and plexin‐B2 receptors are highly expressed on astrocytes intimately associated with neurons in brain.
3. Binding of recombinant soluble SEMA4D to receptors on purified human astrocytes in culture triggers morphological changes along with reduced expression of key metabolic transporters and enzymes that are characteristic of the reactive state. Importantly, this suggests that the SEMA4D:plexin‐B1/B2 pathway regulates not only the actin cytoskeleton but a cascade of neuroinflammatory changes. SEMA4D blocking‐antibody prevents these effects.^4^
4. Because of the important role of astrocytes in glucose transport, we investigated the effect of treatment with our humanised anti‐SEMA4D antibody, pepinemab, on Fluorodeoxyglucose–positron‐emission tomography (FDG‐PET) in different brain regions of patients with early manifest HD, based on diagnostic confidence level and total functional capacity score of 11–13 (top of the scale). As previously reported for HD and other slowly progressive neurodegenerative diseases, FDG‐PET SUVR declines over 18 months progression across almost all brain regions of patients treated with placebo. In striking contrast, pepinemab treatment significantly increased FDG‐PET signal in most cortical regions but, importantly, not in striatum (caudate and putamen, ^3^) of participants with early manifest disease symptoms.

Decline in FDG‐PET has been variously attributed either to reduced glucose uptake by reactive glia (mostly astrocytes) or to loss of energy intensive synaptic activity due to neurodegeneration. We suggest that under conditions in which astrocytes undergo widespread changes between homeostatic and reactive states, decline in FDG‐PET SUVR as observed in cortical regions and its reversal by pepinemab treatment is due to effects on SEMA4D‐dependent astrocyte reactivity. However, in striatum, a brain region known to undergo degeneration early during HD‐progression at a rate approximately four times greater than other brain regions,^6^ it appears that decline in FDG‐PET SUVR may be predominantly associated with neurodegeneration due to direct effects of toxic forms of mutant huntingtin that are not SEMA4D‐dependent and, therefore, not reversed by pepinemab. This suggests a two‐stage model of the neurodegenerative pathology. An initial mutant huntingtin‐dependent (or, in AD, Aβ amyloid or Tau‐dependent) phase that damages neurons and leads to upregulation of SEMA4D followed by a SEMA4D‐dependent neuroinflammatory phase that significantly amplifies and aggravates damage.

## 1 COGNITION

In multiple surveys, HD patients and their families have identified cognitive decline as their major concern during disease progression.^7^, ^8^ The Huntington's Disease Cognitive Assessment Battery (HD‐CAB) is based on six different measures of change in different cognitive domains and has been employed by HD investigators to assess cognitive decline.^9^ The Phase 2 study of pepinemab antibody in Huntington's disease (SIGNAL) study was designed to employ HD‐CAB as a primary cognitive endpoint. We were, however, encouraged by FDA to instead adopt a novel two‐item cognitive family comprised of the one touch stockings (OTS) and paced tapping (PTAP) measures from HD‐CAB, primarily, it seemed, to avoid use of a composite score of six assessments. Unfortunately, the SIGNAL study was not designed to be powered for this substitute endpoint and the effect of pepinemab treatment on OTS just missed significance (one‐sided *p* = .028) and for PTAP only showed a trend (*p* = .06). As originally planned, however, the study was well‐powered for HD‐CAB, retained as an exploratory endpoint, and indicated a highly significant treatment effect (*p* = .007). This was consistent with significantly reduced apathy severity (*p* = .017) which several studies in HD and AD have shown to be correlated with cognition .^10^ Similarly, multiple studies have reported that decline in FDG‐PET correlates with cognitive decline during disease progression in AD.^11^, ^12^ Pepinemab is, to our knowledge, the only agent that has been shown to reverse both metabolic and cognitive decline in a neurodegenerative disease.

**FIGURE 1:**
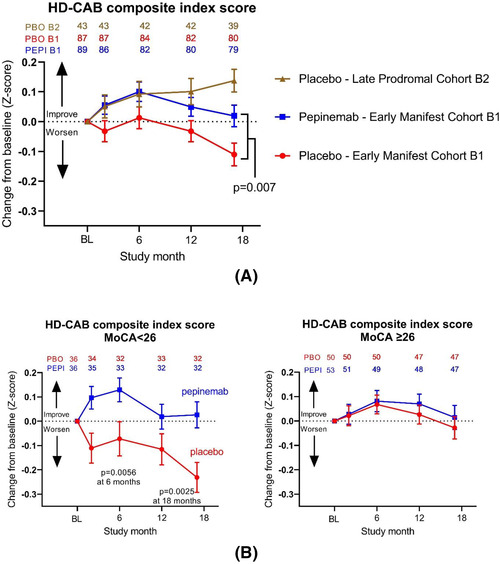
(A) Disease progression impedes and pepinemab treatment restores significant “Learning” effects detected by Huntington's Disease Cognitive Assessment Battery (HD‐CAB). *Note*: no significant change is observed in late prodromal group treated with pepinemab as reported in *Nature Med*. 2022;28(10):2183‐2193. (B) Significant HD‐CAB treatment effects as early as 6 months in the HD patient subpopulation with early signs of cognitive deficits (MoCA < 26)

As previously reported for AD, onset of symptomatic disease is associated with loss of “learning effects”.^13^, ^14^ It is, therefore, of particular interest that patients with early manifest HD do not show the improvement in performance on the sequential administration of HD‐CAB that is evident in largely cognitively normal (83% MoCA ≥ 26) late prodromal subjects (Figure 1A). Importantly, the ability to learn from experience is restored and HD‐CAB performance improves in early manifest HD patients during the first 6 months of pepinemab treatment after which continued disease progression appears to impact performance. We suggest that “learning” is intrinsically significant to patients and could serve as meaningful evidence of clinical benefit. This is an important consideration for design of the next phase 3 study in HD.

In view of the two‐stage model of neurodegenerative pathology suggested by differential effects of pepinemab treatment on FDG‐PET in cortical regions versus striatum during early HD progression, we considered whether cognitive treatment effects might be most evident in patients with early evidence of cognitive decline. Analysis of the effect of treatment on HD‐CAB was, therefore, stratified by the Montreal cognitive assessment (MoCA) score at baseline. As seen in Figure 1B, a significant treatment effect (*p* = .056) was observed in the MoCA < 26 subgroup (cognitive deficit) during the first 6 months of the treatment but was not discernible in the MoCA ≥ 26 subgroup (normal cognition).

## 2 ALZHEIMER's DISEASE

Evidence of neuroinflammation suggests that the mechanism of action of pepinemab is relevant to pathogenesis of AD and other slowly progressive neurodegenerative diseases (e.g., Parkinson's and progressive Multiple Sclerosis (MS)) as well as HD. There are, of course, important differences among these diseases in the specific stress‐inducing event that initiates pathology and the brain regions affected. Early degeneration in AD appears to center on the entorhinal cortex rather than the striatum as in HD. An early phase SIGNAL‐AD study enrolling patients with mild‐AD is currently in progress. Pepinemab could be a promising therapeutic as a single agent, as was observed in HD. Additionally, it could be employed in combination therapy to augment efficacy and possibly reduce disease related neuroinflammatory effects.

## CONFLICT OF INTEREST

The authors are employees of and own stock or stock options in Vaccinex, Inc.

## References

[1] Robinson MB , Jackson JG . Astroglial glutamate transporters coordinate excitatory signaling and brain energetics. Neurochem Int. 2016;98:56‐71. 27013346 10.1016/j.neuint.2016.03.014 PMC4969184

[2] Liddelow SA , Guttenplan KA , Clarke LE , et al. Neurotoxic reactive astrocytes are induced by activated microglia. Nature. 2017;541:481‐487. 28099414 10.1038/nature21029 PMC5404890

[3] Feigin A , Evans EE , Fisher TL , et al. Pepinemab antibody blockade of SEMA4D in early Huntington's disease: a randomized, placebo‐controlled, phase 2 trial. Nature Med. 2022;28:2183‐2193. 35941373 10.1038/s41591-022-01919-8 PMC9361919

[4] Evans EE , Mishra V , Mallow C , et al. Semaphorin 4D is upregulated in neurons of diseased brains and triggers astrocyte reactivity. J Neuroinflammation. 2022;19:200. 35933420 10.1186/s12974-022-02509-8 PMC9356477

[5] Basile JR , Gavard J , Gutkind JS . Plexin‐B1 utilizes RHOA and ROK to promote the integrin‐dependent activation of AKT and ERK, and endothelial cell motility. J Biol Chem. 2007;282:34888‐34895. 17855350 10.1074/jbc.M705467200

[6] Johnson EB , Ziegler G , Penny W , et al. Dynamics of cortical degeneration over a decade in Huntington's disease. Biol Psychiatry. 2021;89:807‐816. 33500176 10.1016/j.biopsych.2020.11.009 PMC7986936

[7] Tabrizi SJ , Scahill RI , Owen G , et al. Predictors of phenotypic progression and disease onset in premanifest and early‐stage Huntington's disease in the TRACK‐HD study: analysis of 36‐month observational data. Lancet Neurol. 2013;12:637‐649. 23664844 10.1016/S1474-4422(13)70088-7

[8] The Voice of the Patient : Huntington's Disease. Food and Drug Administration. Patient Focused Drug Development Initiative. FDA; 2016.

[9] Stout JC , Queller S , Baker KN , et al. HD‐CAB: a cognitive assessment battery for clinical trials in Huntington's disease. Mov Disord. 2014;29(10):1281‐1288. 25209258 10.1002/mds.25964

[10] Baudic S , Langbehn DR , Craufurd D , et al. Cognitive impairment related to apathy in early Huntington's disease. Dement Geriatr Cogn Disord. 2006;21:316‐321. 16484810 10.1159/000091523

[11] Landau SM , Harvey D , Madison CM , et al. Associations between cognitive, functional, and FDG‐PET measures of decline in AD and MCI. Neurobiol Aging. 2011;32:1207‐1218. 19660834 10.1016/j.neurobiolaging.2009.07.002 PMC2891865

[12] Hanseeuw BJ , Betensky RA , Schultz AP , et al. Fluorodeoxyglucose metabolism associated with tau‐amyloid interaction predicts memory decline. Ann Neurol. 2017;81:583‐596. 28253546 10.1002/ana.24910 PMC5404378

[13] Samaroo A , Amariglio RE , Burnham S , et al. Diminished learning over repeated exposures (LORE) in preclinical Alzheimer's disease. Alzheimers Dement. 2020;12:e12132. 10.1002/dad2.12132 PMC7784542 33426266

[14] utten RJ , Rentz DM , Fu JF , et al. Monthly at‐home computerized cognitive testing to detect diminished practice effects in preclinical Alzheimer's disease. *Front* Aging Neurosci. 2021;13:800126. 10.3389/fnagi.2021.800126 PMC8792465 35095476

